# Genetic Diversity of Highly Pathogenic Avian Influenza A(H5N8/H5N5) Viruses in Italy, 2016–17

**DOI:** 10.3201/eid2309.170539

**Published:** 2017-09

**Authors:** Alice Fusaro, Isabella Monne, Paolo Mulatti, Bianca Zecchin, Lebana Bonfanti, Silvia Ormelli, Adelaide Milani, Krizia Cecchettin, Philippe Lemey, Ana Moreno, Paola Massi, Tiziano Dorotea, Stefano Marangon, Calogero Terregino

**Affiliations:** Istituto Zooprofilattico Sperimentale delle Venezie, Legnaro, Italy (A. Fusaro, I. Monne, P. Mulatti, B. Zecchin, L. Bonfanti, S. Ormelli, A. Milani, K. Cecchettin, T. Dorotea, S. Marangon, C. Terregino);; Rega Institute, KU Leuven, Leuven, Belgium (P. Lemey);; Istituto Zooprofilattico Sperimentale della Lombardia e dell’Emilia Romagna, Brescia, Italy (A. Moreno, P. Massi).

**Keywords:** influenza, influenza A virus, H5N8 subtype, H5N5 subtype, phylogeny, reassortments, Italy, viruses, respiratory infections, zoonoses, highly pathogenic avian influenza

## Abstract

In winter 2016–17, highly pathogenic avian influenza A(H5N8) and A(H5N5) viruses of clade 2.3.4.4 were identified in wild and domestic birds in Italy. We report the occurrence of multiple introductions and describe the identification in Europe of 2 novel genotypes, generated through multiple reassortment events.

In spring 2016, highly pathogenic avian influenza (HPAI) outbreaks caused by the H5N8 subtype of clade 2.3.4.4 (group B) were reported in migratory wild birds in Qinghai Lake, China (*1*), and in the salt lake system of Uvs Nuur on the Russian Federation–Mongolia border (*2*). Since then, HPAI A(H5N8) viruses have been detected in several countries in Asia, Europe, and Africa. In Europe, the virus was detected for the first time in October 2016 in Hungary (*3*). Here, we describe the occurrence of multiple introductions of reassortant HPAI A(H5N8) and A(H5N5) viruses in Italy, in both wild and domestic birds.

## The Study

**Figure 1:**
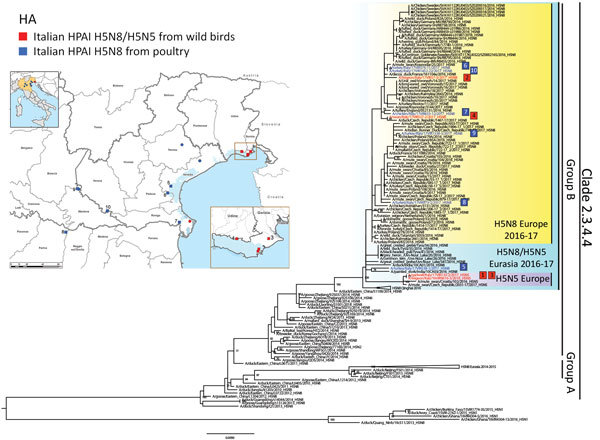
Highly pathogenic avian influenza A(H5N8) and A(H5N5) in birds, Italy, 2016–17). A) Geographic distribution of cases in wild (red) and domestic (blue) birds in northern Italy. Squares indicate the samples sequenced in this study; circles indicate positive samples for which no genetic information was available at the time of writing. B) Maximum likelihood phylogenetic tree of the hemagglutinin gene of clade 2.3.4.4 viruses. Viruses analyzed in this study are indicated with red (wild birds) and blue (domestic birds) squares, numbered according to the collection date. Bootstrap supports >60% are indicated above the nodes. Scale bar indicates nucleotide substitutions per site.

During December 2016–January 2017, a Eurasian wigeon (*Anas penelope*) and a gadwall (*Anas strepera*) found dead at Grado Lagoon in northeastern Italy tested positive for HPAI A(H5N5). A second wigeon tested positive for HPAI A(H5N8). Since then, additional HPAI A(H5N8) cases were observed in a common shelduck (*Tadorna tadorna*) and in a mute swan (*Cygnus olor*) and in birds on 6 commercial turkey farms, 1 layer farm, and 3 backyard flocks (Table 1; Figure 1). All of the cases in domestic poultry farms occurred in areas in close proximity to wetlands that are listed as important resting sites for migratory waterfowl. The onset of clinical signs in all the affected poultry species was generally associated with depression, reluctance to move, and a drop in feed consumption. The clinical condition often evolved into a more severe respiratory and nervous syndrome associated with an increased mortality rate (average mortality rate is 1.62% [95% CI 1.10%–2.14%]). Depopulation measures on the infected farms and 7 neighboring poultry premises considered at risk involved ≈510,000 birds.

**Table 1:** Epidemiologic information for highly pathogenic avian influenza A(H5N5) and A(H5N8) viruses isolated from birds in Italy, 2016–17

Isolate	Type	Collection date	Region	Location	Site type	EpiFlu accession no.*
A/wigeon/Italy/16VIR9616-3/2016	H5N5	2016 Dec 29	Friuli Venezia Giulia	Grado (Gorizia)	Natural park	EPI888600-01, EPI954800-05
A/wigeon/Italy/17VIR57-3/2017	H5N8	2017 Jan 03	Friuli Venezia Giulia	Grado (Gorizia)	Natural park	EPI888085-92
A/gadwall/Italy/17VIR133-2/2017	H5N5	2017 Jan 10	Friuli Venezia Giulia	Grado (Gorizia)	Natural park	EPI954616-23
A/swan/Italy/17VIR537-2/2017	H5N8	2017 Jan 19	Friuli Venezia Giulia	Aquileia (Udine)	Natural park	EPI954552-59
A/turkey/Italy/17VIR538-1/2017	H5N8	2017 Jan 20	Veneto	Mira (Venice)	Fattening turkeys farm	EPI954560-67
A/turkey/Italy/17VIR576-11/2017	H5N8	2017 Jan 23	Veneto	Piove di Sacco (Padua)	Fattening turkeys farm	EPI954568-75
A/chicken/Italy/17VIR653-12/2017	H5N8	2017 Jan 25	Veneto	Porto Viro (Rovigo)	Laying hens farm	EPI954576-83
A/turkey/Italy/17VIR973-2/2017	H5N8	2017 Feb 01	Emilia Romagna	Sorbolo (Parma)	Fattening turkeys farm	EPI954584-91
A/turkey/Italy/17VIR1338-3/2017	H5N8	2017 Feb 14	Lombardy	Monzambano (Mantova)	Fattening turkeys farm	EPI954592-99
A/turkey/Italy/17VIR1452-22/2017	H5N8	2017 Feb 16	Veneto	Gazzo Veronese (Verona)	Fattening turkeys farm	EPI954600-07

*GISAID EpiFlu database (http://platform.gisaid.org).

**Figure 2:**
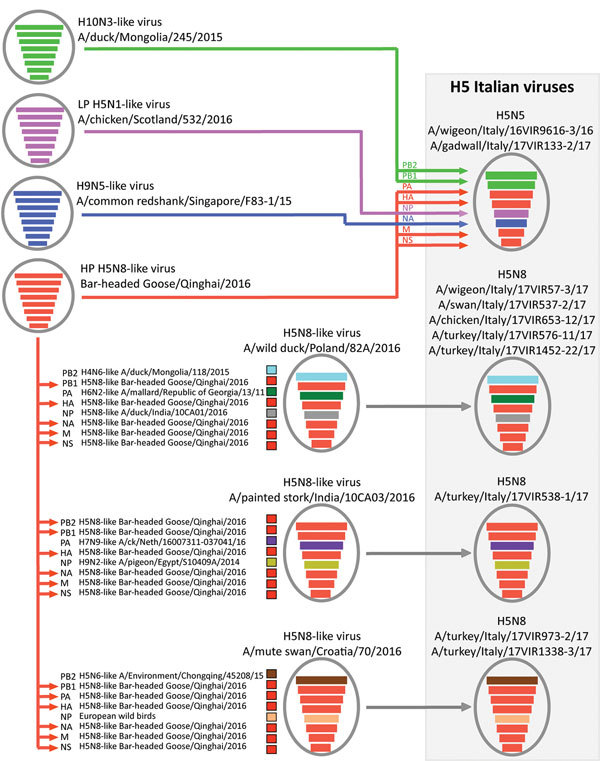
Probable genesis of highly pathogenic avian influenza A(H5N8) and A(H5N5) reassortant viruses identified in Italy, 2016–17 (gray box). Virus particles are represented by ovals containing horizontal bars that represent the 8 gene segments, colored according to their origin.

The genomes of 10 positive samples collected from wild (n = 4) and domestic (n = 6) birds were fully sequenced (Technical Appendix 1). Phylogenetic analysis of the hemagglutinin (HA) gene showed that the HPAI A(H5N5) and A(H5N8) viruses clustered within the 2.3.4.4 clade, group B (Figure 1). However, the characterization of the complete genome (Technical Appendix 1 Figures 1–8) revealed that these viruses belong to 4 distinct genotypes, which had very likely originated from multiple reassortment events.

Phylogenetic analyses indicated that the HPAI H5N5 viruses had been generated through intersubtype reassortment events between the H5N8 viruses from Asia (H5N8-Gs/Qinghai/2016-like) and the low pathogenicity avian influenza (LPAI) viruses of the Eurasian lineage (Figure 2). The A(H5N8) viruses from Asia were the source of the HA, polymerase acidic, matrix, and nonstructural protein genes. HPAI A(H5N5) viruses with similar HA and neuraminidase genes were identified in Croatia and Czech Republic in 2016–17. The time to the most recent common ancestor (tMRCA) estimated by pooling the information across all the gene segments in a hierarchical model (Technical Appendix 1) suggested that a virus with this gene constellation emerged during October–December 2016 (Table 2; Technical Appendix 1 Table 1).

**Table 2:** tMRCA for the 4 avian influenza A(H5N5) and A(H5N8) virus genotypes identified in Italy, 2016–17*

Genotype	tMRCA	
	Mean	95% HPD
H5N5	November 2016	October–December 2016
H5N8 A/wild duck/Poland/82A/2016-like	May 2016	May–June 2016
H5N8 A/painted stork/India/10CA03/2016-like	August 2016	July–October 2016
H5N8 A/mute swan/Croatia/70/2016-like	July 2016	June–August 2016

*tMRCAs estimated for each gene segments are reported in Technical Appendix 1 Table 1. HPD, highest posterior density; tMRCA, time to most recent common ancestor.

Among the 8 HPAI A(H5N8) viruses in Italy investigated during this study, 5 were collected from wild and domestic birds in the Veneto region. In all the phylogenetic trees, these viruses clustered within the main European A(H5N8) group (A/wild duck/Poland/82A/2016-like) (Figure 2), previously described by Pohlmann et al. (*4*). The tMRCA for this group was May–June 2016 in the hierarchical gene segment model (Table 2; Technical Appendix 1 Table 1). The first HPAI A(H5N8) virus detected in a turkey farm in the Veneto region displayed the gene composition of a virus isolated in October 2016 from a painted stork in an Indian zoo (*5*), which had not previously been reported in Europe (Figure 2). The tMRCA of this Indian–Italian group is July–October 2016, according to the hierarchical gene segment model (Table 2; Technical Appendix 1 Table 1). The 2 outbreaks reported in 2 commercial turkey farms in the Emilia-Romagna and Lombardy regions were caused by HPAI A(H5N8) reassortant viruses containing the polymerase basic protein 2 and nucleoprotein genes of LPAI viruses of the Eurasian lineage and the remaining genes from the H5N8-Gs/Qinghai/2016-like genotype (Figure 2). Viruses with a similar gene pool were identified in Croatia and France. Estimation of the tMRCA by the hierarchical gene segment model indicated that this genotype might have emerged during June–August 2016 (Table 2; Technical Appendix 1 Table 1).

Analyses of the phylogenetic topologies revealed that most of the sequences found in Italy were dispersed throughout the trees, indicating the occurrence of several independent introductions of the A(H5N8) virus into poultry farms from wild birds (Technical Appendix 1 Figures 1–8). These results were confirmed by our median-joining network analyses for the HA gene (Technical Appendix 1 Figure 9), which showed that the ancestral sequences of the samples from Italy represent viruses collected in other countries. In most cases >1 median vector, representing the lost ancestral sequences, separated these viruses from the hypothetical progenitor. The only exception was for A/turkey/Italy/17VIR576-11/2017 and A/turkey/Italy/17VIR1452-22/2017, which proved to be almost identical for all the genes (similarity of 99.9%–100%), although they were collected 24 days apart in 2 turkey flocks located at a distance of ≈90 km from one another and no evident contacts were observed between them. However, because the 2 outbreaks had occurred in 2 farms operated by the same company, an exchange of virus cannot be ruled out.

Intravenous pathogenicity indexes obtained for 8 representative A(H5N8) and A(H5N5) isolates ranged from 2.85–3, comparable to an index of 2.93 for 2016 A(H5N8) viruses from Germany and 2.75–2.84 for 2016 A(H5N8) viruses from Russia (*2**,**4*). These data confirm that both of the A(H5N8) and A(H5N5) viruses from Italy, which shared the same HA cleavage site (PLREKRRKR), are highly pathogenic for poultry.

## Conclusions

Since its emergence in China in 2013, the HPAI H5 of clade 2.3.4.4 has evolved in different genetic groups, namely A to D (*6*). Here, we describe the introductions of 4 different H5 viral genotypes of clade 2.3.4.4 group B in northern Italy. As previously observed for the 2014–15 A(H5N8) epidemic wave (*7*), our results confirm that these strains have a high propensity to reassort with co-circulating LPAI and HPAI viruses, causing the generation of several subtypes and genotypes with unique gene constellations. Unfortunately, the lack of sequences of the potential progenitors, exemplified by the long branches observed in particular in the polymerase basic protein 2, polymerase acidic, and nucleoprotein phylogenies, makes it difficult to determine when and where these genotypes emerged. The genetic variability observed in the viruses identified in domestic birds, the similarity to viruses circulating in Europe and India, and the close proximity of the infected poultry farms to wetlands all suggest that wild birds did play a major role in the multiple and independent introductions of the virus into poultry holdings.

Our study highlights the importance of generating complete viral genome sequences in a timely fashion, which may help to monitor the viral spread and define appropriate disease control strategies. This, coupled with intensified wild bird surveillance on wetlands of ecologic importance for avian influenza viruses, can improve our understanding of the virus dissemination routes and support early detection of viruses highly pathogenic to poultry or believed to be of immediate concern to human health.

Technical Appendix 1 Methods and additional results in a study of highly pathogenic avian influenza A(H5N8) and A(H5N5) in birds in Italy .

Technical Appendix 2 GISAID database isolates used in this study.
