# Supplementary material for: Genetic Diversity of Highly Pathogenic Avian Influenza A(H5N8/H5N5) Viruses in Italy, 2016–17
Source: Emerg Infect Dis. 2017 Sep;23(9):1543–7. doi: 10.3201/eid2309.170539 (PMC5572881; doi:10.3201/eid2309.170539)
Supplement: Technical Appendix 1 — Methods and additional results in a study of highly pathogenic avian influenza A(H5N8) and A(H5N5) in birds in Italy . [file 17-0539-Techapp-s1.pdf]

Article DOI: https://doi.org/10.3201/eid2309.170539

Genetic Diversity of Highly Pathogenic Avian Influenza A(H5N8/H5N5) Viruses in Italy, 2016–17

Technical Appendix 1

Materials and Methods

Genome sequencing

Total RNA was purified from 8 HPAI H5N8 and 2 HPAI H5N5 positive clinical samples using the Nucleospin RNA kit (Macherey–Nagel, Duren, Germany). Complete influenza A virus genomes were amplified with the SuperScript III One-Step RT-PCR system with Platinum Taq High Fidelity (Invitrogen, Carlsbad, CA) using one pair of primers complementary to the conserved elements of the influenza A virus promoter as described in (*1*). Sequencing libraries were obtained using Nextera DNA XT Sample preparation kit (Illumina) following the manufacturer's instructions and quantified using the Qubit dsDNA High Sensitivity kit (Invitrogen, USA). The average fragment length was determined using the Agilent High Sensitivity Bioanalyzer Kit. The indexed libraries were pooled in equimolar concentrations and sequenced in multiplex for 250 bp paired-end on Illumina MiSeq, according to the manufacturer's instructions.

High-throughput sequencing data analysis

Illumina reads quality was assessed using FastQC v0.11.2. Raw data were filtered by removing: i) reads with more than 10% of undetermined ("N") bases; ii) reads with more than 100 bases with Q score below 7; iii) duplicated paired-end reads. Remaining reads were clipped from Illumina Nextera XT adaptors with scythe v0.991 (https://github.com/vsbuffalo/scythe) and trimmed with sickle v1.33 (https://github.com/najoshi/sickle). Reads shorter than 80 bases or unpaired after previous filters were discarded. High-quality reads were aligned against a reference genome using BWA v0.7.12 (*2*). Alignments were processed with Picard-tools v2.1.0 (http://picard.sourceforge.net) and GATK v3.5 (*3*–*5*) to correct potential errors, realign reads

around indels, and recalibrate base quality. Single Nucleotide Polymorphisms (SNPs) were called using LoFreq v2.1.2 (*6*) and the outputs were used to generate the consensus sequences.

Phylogenetic analyses

Consensus sequences of the complete genome of the 10 samples were aligned using MAFFT v. Seven (*7*) and compared with the most related sequences available in GISAID (accessed February 28, 2017). Maximum likelihood (ML) phylogenetic trees were obtained for each gene segment using the best-fit general time-reversible (GTR) model of nucleotide substitution with gamma-distributed rate variation among sites (with 4 rate categories, Γ4) and a heuristic SPR branch-swapping search (*8*) available in the PhyML program version 3.1. To assess the robustness of individual nodes of the phylogeny, 100 bootstrap replicates were performed. Phylogenetic trees were visualized with the program FigTree v1.4.2 (http://tree.bio.ed.ac.uk/software/figtree/).

The HA gene segment of the HPAI H5N8 and H5N5 influenza viruses collected in Eurasia in 2016–2017 was aligned and used to construct a phylogenetic network using the Median Joining method implemented in the program NETWORK 4.5 (http://www.fluxus-engineering.com) (*9*). This method uses a parsimony approach to reconstruct the relationships between highly similar sequences, and allows the creation of "median vectors," which represent unsampled sequences that are used to connect the existing genotypes in the most parsimonious way. The parameter *epsilon* was set to 0.

Estimation of the Time to the Most Recent Common Ancestor (tMRCA)

We estimated the tMRCAs of the HPAI H5N8 and H5N5 genotypes identified in Italy by applying a Bayesian hierarchical model to all 8 gene segments using the BEAST software (*10*). To model the substitution process in each gene segment, we employed an HKY85 + Γ_4 model with two partitions (1st + 2nd positions versus 3rd position), base frequencies and Γ-rate heterogeneity unlinked across all codon positions (the SRD06 substitution model). We specified an independent uncorrelated lognormal relaxed clock and Bayesian skyride tree prior for each segment. To allow pooling of information across segments in estimating the tMRCAs of the HPAI H5N8 and H5N5 genotypes, we specified a hierarchical prior distribution over each of the corresponding tMRCAs of the genotypes (*11*). Specifically, we assume that the log of the tMRCAs are drawn from a normal distribution with a mean and a variance that is also unknown

and simultaneously estimated along with all the sequence evolution parameters. We used Markov chain Monte Carlo (MCMC) to draw inference under this model and used chain lengths of 50 million iterations to achieve convergence as assessed using Tracer v1.6 (http://beast.bio.ed.ac.uk/Tracer). Maximum Clade Credibility (MCC) phylogenetic trees were summarized from the posterior distribution of trees using TreeAnnotator v1.6.1 (*10*) after the removal of an appropriate burn-in (10% of the samples). The MCC trees were visualized using the program FigTree v1.4.2 (http://tree.bio.ed.ac.uk/software/figtree/).

References

1. Zhou B, Donnelly ME, Scholes DT, St. George K, Hatta M, Kawaoka Y, et al. Single-reaction genomic amplification accelerates sequencing and vaccine production for classical and swine origin human influenza A viruses. J Virol. 2009;83:10309–13.

2. Li H, Durbin R. Fast and accurate long-read alignment with Burrows-Wheeler transform. Bioinformatics. 2010;26:589–95.

3. McKenna A, Hanna M, Banks E, Sivachenko A, Cibulskis K, Kernytsky A, et al. The Genome Analysis Toolkit: a MapReduce framework for analyzing next-generation DNA sequencing data. Genome Res. 2010;20:1297–303.

4. DePristo MA, Banks E, Poplin R, Garimella KV, Maguire JR, Hartl C, et al. A framework for variation discovery and genotyping using next-generation DNA sequencing data. Nat Genet. 2011;43:491–8.

5. Van der Auwera GA, Carneiro MO, Hartl C, Poplin R, Del Angel G, Levy-Moonshine A, et al. From FastQ data to high confidence variant calls: the Genome Analysis Toolkit best practices pipeline. Curr Protoc Bioinformatics. 2013;43:11.10.1–33.

6. Wilm A, Aw PP, Bertrand D, Yeo GH, Ong SH, Wong CH, et al. LoFreq: a sequence-quality aware, ultra-sensitive variant caller for uncovering cell-population heterogeneity from high-throughput sequencing datasets. Nucleic Acids Res. 2012;40:11189–201.

7. Katoh K, Standley DM. MAFFT multiple sequence alignment software version 7: improvements in performance and usability. Mol Biol Evol. 2013;30:772–80.

8. Guindon S, Gascuel O. A simple, fast, and accurate algorithm to estimate large phylogenies by maximum likelihood. Syst Biol. 2003;52:696–704.

9. Bandelt HJ, Forster P, Röhl A. Median-joining networks for inferring intraspecific phylogenies. Mol Biol Evol. 1999;16:37–48.

10. Drummond AJ, Rambaut A. BEAST: Bayesian evolutionary analysis by sampling trees. BMC Evol Biol. 2007;7:214.

11. Edo-Matas D, Lemey P, Tom JA, Serna-Bolea C, van den Blink AE, van 't Wout AB, et al. Impact of CCR5delta32 host genetic background and disease progression on HIV-1 intrahost evolutionary processes: efficient hypothesis testing through hierarchical phylogenetic models. Mol Biol Evol. 2011;28:1605–16.

Technical Appendix Table 1. Time to the most recent ancestor (tMRCA) for each gene segment of the 4 genotypes identified in Italy

	Genotype							
	H5n5		H5n8 A/Wild Duck/Poland/82a/16-Like		H5n8 A/Painted Stork/India/10ca03/16-Like		H5n8 A/Mute Swan/Croatia/70/16-Like	
Gene	Mean	95% Hpd	Mean	95% Hpd	Mean	95% Hpd	Mean	95% Hpd
PB2	Nov 2016	Oct–Dec 2016	May 2016	May–Jun 2016	Sep 2016	Jul–Oct 2016	Jul 2016	Jun–Sep 2016
PB1	Nov 2016	Oct–Dec 2016	May 2016	May–Jun 2016	Aug 2016	Jul–Oct 2016	Jul 2016	Jun–Aug 2016
PA	Nov 2016	Sep–Dec 2016	May 2016	May–Jun 2016	Aug 2016	Jul–Oct 2016	Jul 2016	Jun–Aug 2016
HA	Nov 2016	Oct–Dec 2016	Jun 2016	May–Jun 2016	Aug 2016	Jul–Sep 2016	Jul 2016	Jun–Sep 2016
NP	Nov 2016	Oct–Dec 2016	May 2016	May–Jun 2016	Aug 2016	Jul–Oct 2016	Jul 2016	Jun–Aug 2016
NA	Nov 2016	Sep–Dec 2016	Jun 2016	May–Jun 2016	Aug 2016	Jul–Oct 2016	Jul 2016	Jun–Aug 2016
M	Nov 2016	Oct–Dec 2016	Jun 2016	May–Jun 2016	Sep 2016	Aug–Oct 2016	Jul 2016	Jun–Aug 2016
NS	Nov 2016	Oct–Dec 2016	Jun 2016	May–Jun 2016	Sep 2016	Aug–Oct 2016	Jul 2016	Jun–Aug 2016

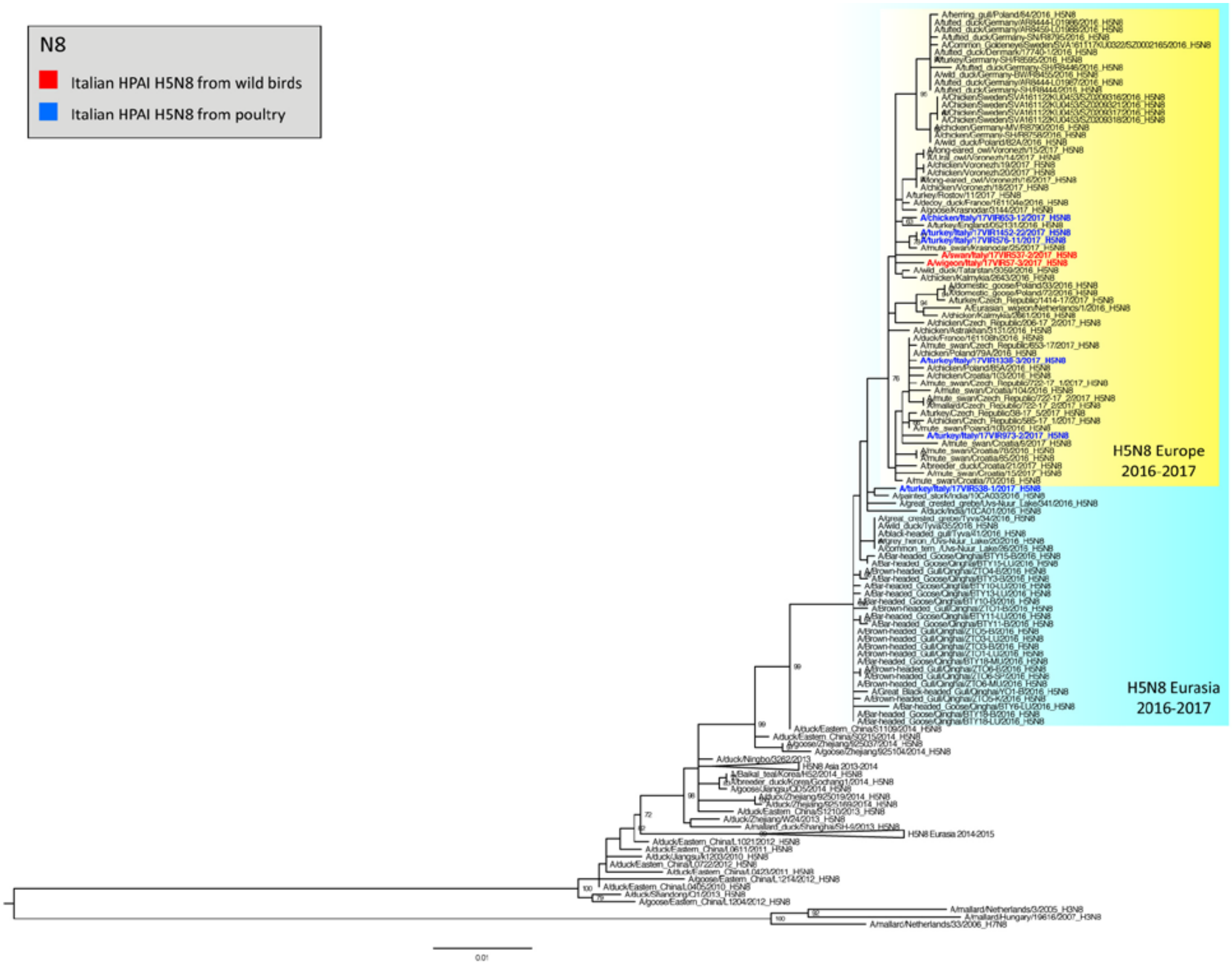

Technical Appendix Figure 1. Maximum Likelihood phylogenetic tree of the NA gene of the N8 subtype. HPAI H5N8 viruses were collected in Italy from wild (red) and domestic (blue) birds. Bootstrap supports higher than 60% are indicated next to the nodes, while branch lengths are scaled according to the number of nucleotide substitutions per site. The tree is midpoint rooted for clarity only.

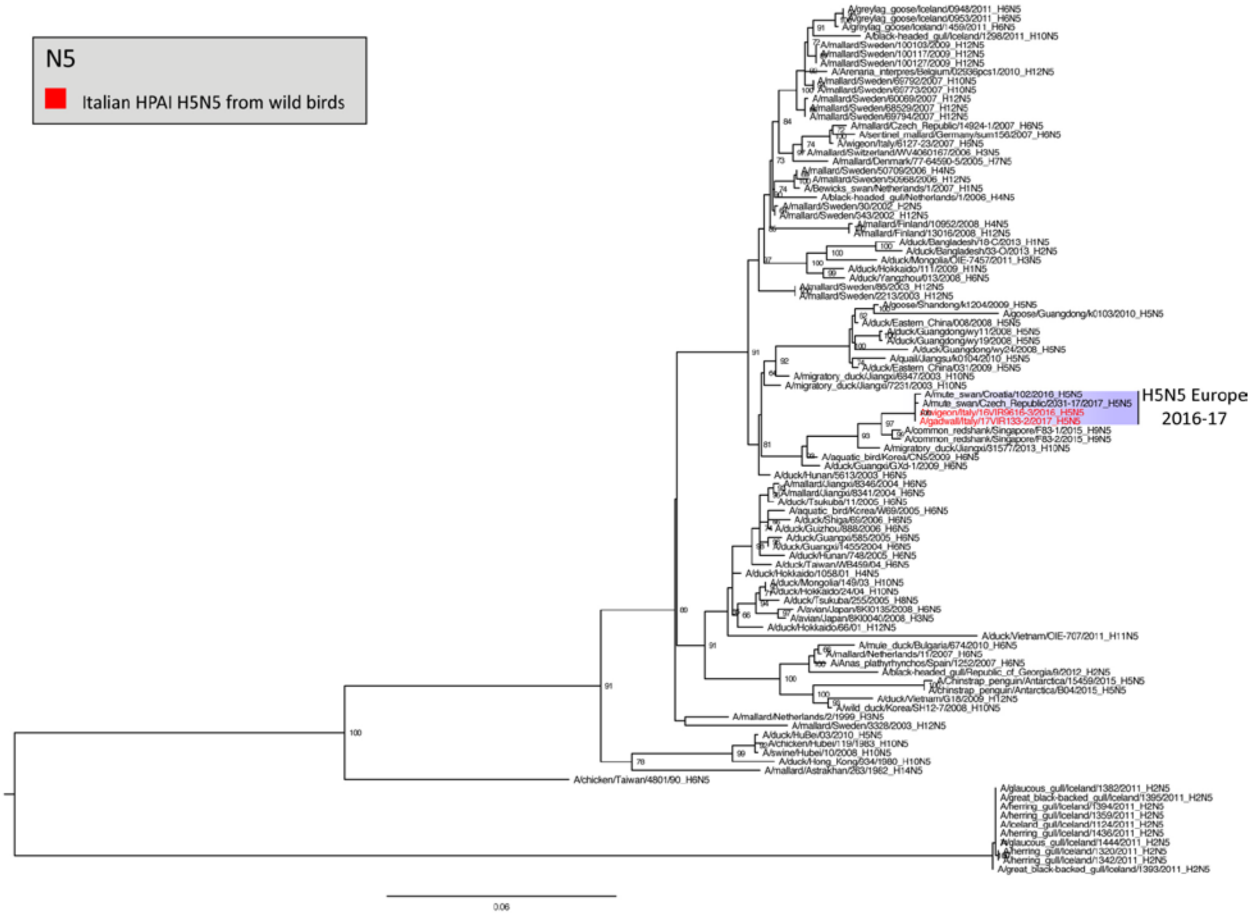

Technical Appendix Figure 2. Maximum Likelihood phylogenetic tree of the NA gene of the N5 subtype. HPAI H5N5 viruses collected in Italy from wild birds are marked in red. Bootstrap supports higher than 60% are indicated next to the nodes, while branch lengths are scaled according to the number of nucleotide substitutions per site. The tree is midpoint rooted for clarity only.

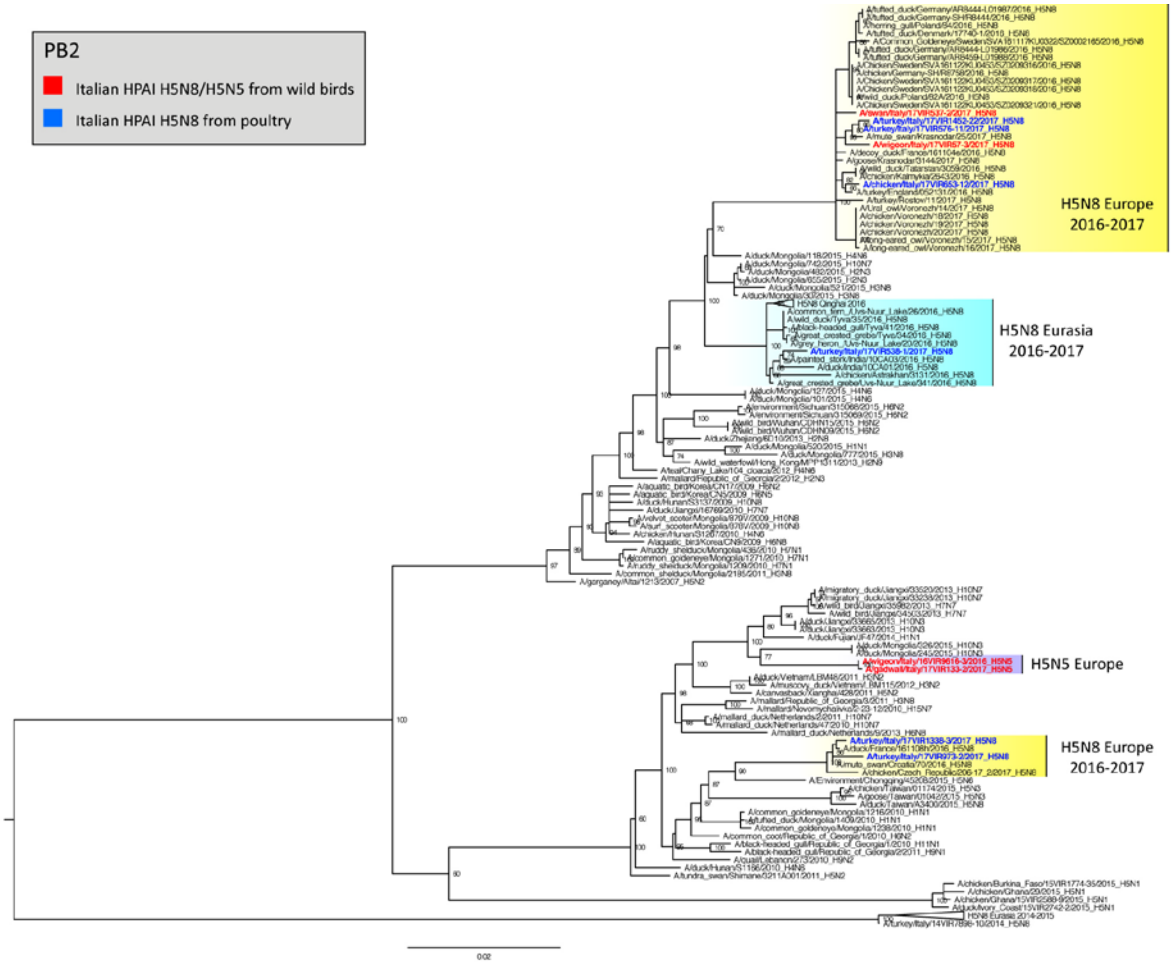

Technical Appendix Figure 3. Maximum Likelihood phylogenetic tree of the PB2 gene. HPAI H5N8/H5N5 viruses were collected in Italy from wild (red) and domestic (blue) birds. Bootstrap supports higher than 60% are indicated next to the nodes, while branch lengths are scaled according to the number of nucleotide substitutions per site. The tree is midpoint rooted for clarity only.

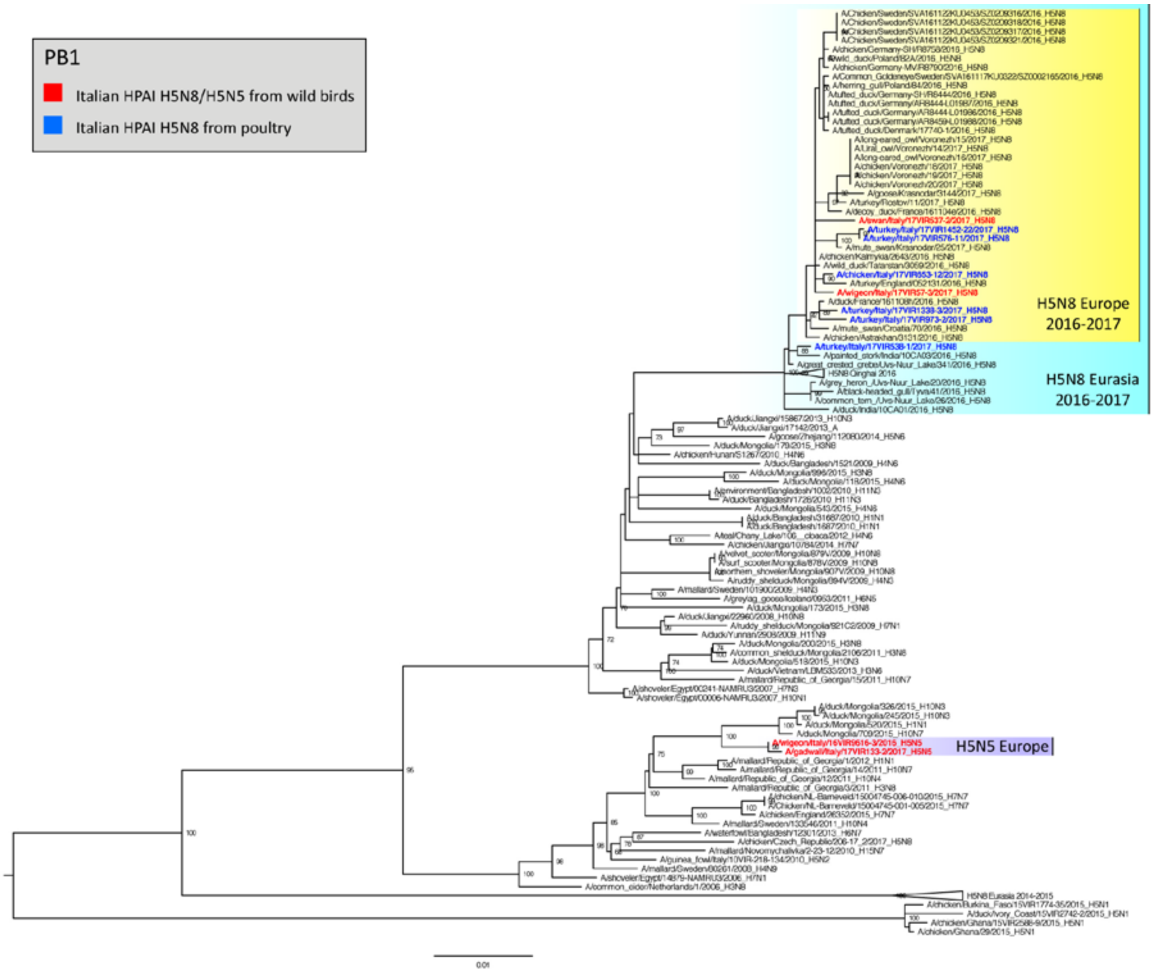

Technical Appendix Figure 4. Maximum Likelihood phylogenetic tree of the PB1 gene. HPAI H5N8/H5N5 viruses were collected in Italy from wild (red) and domestic (blue) birds. Bootstrap supports higher than 60% are indicated next to the nodes, while branch lengths are scaled according to the number of nucleotide substitutions per site. The tree is midpoint rooted for clarity only.

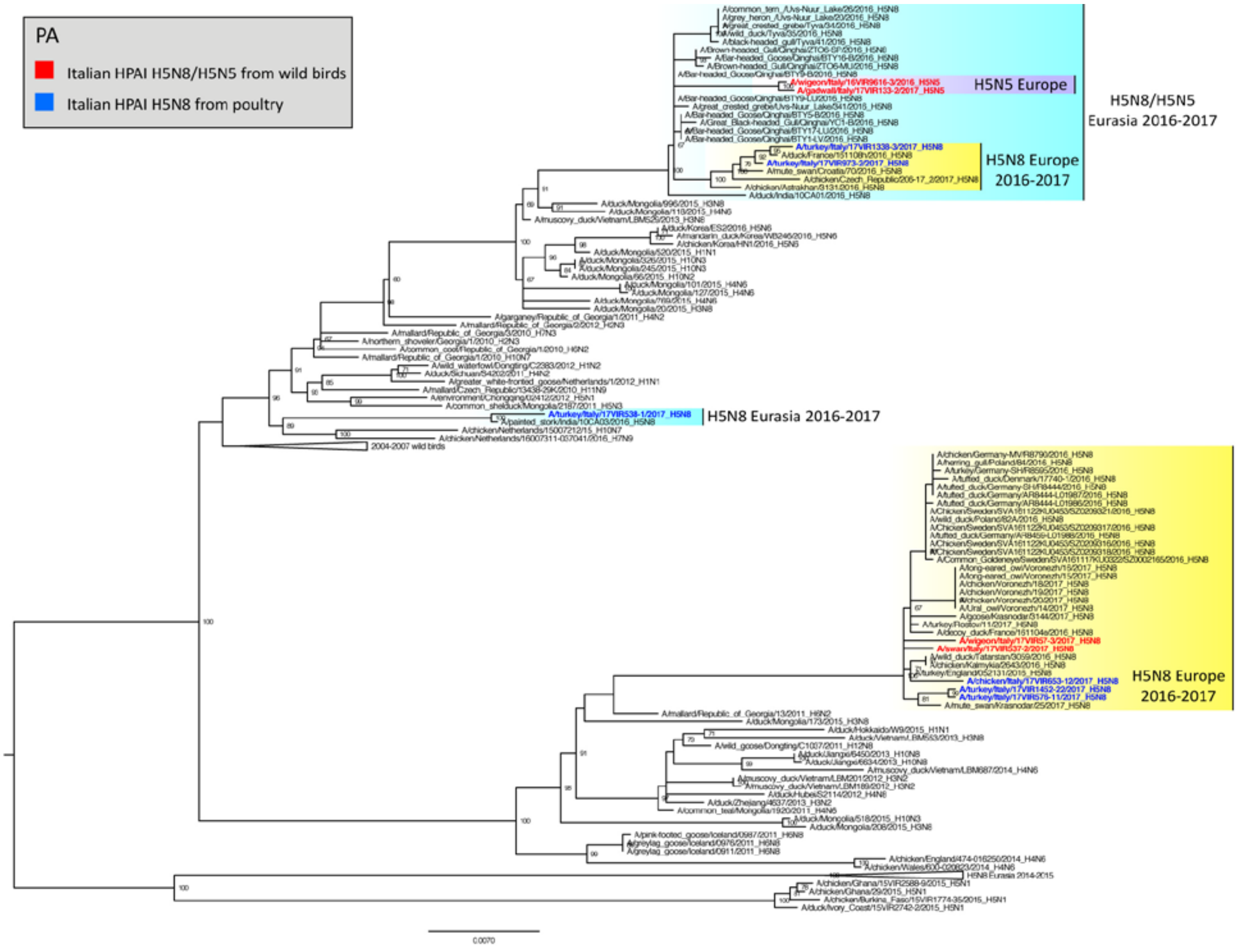

Technical Appendix Figure 5. Maximum Likelihood phylogenetic tree of the PA gene. HPAI H5N8/H5N5 viruses were collected in Italy from wild (red) and domestic (blue) birds. Bootstrap supports higher than 60% are indicated next to the nodes, while branch lengths are scaled according to the number of nucleotide substitutions per site. The tree is midpoint rooted for clarity only.

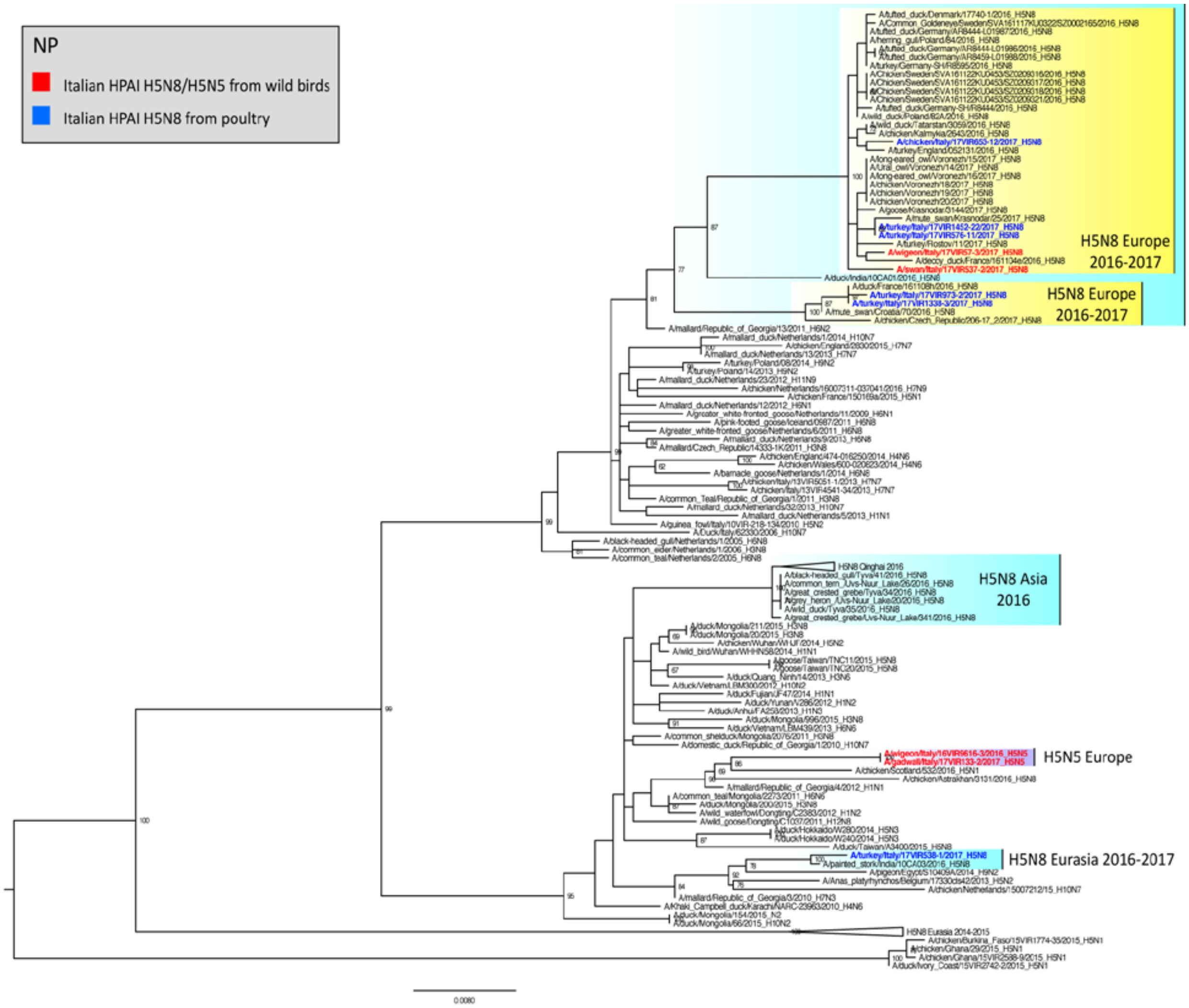

Technical Appendix Figure 6. Maximum Likelihood phylogenetic tree of the NP gene. HPAI H5N8/H5N5 viruses were collected in Italy from wild (red) and domestic (blue) birds. Bootstrap supports higher than 60% are indicated next to the nodes, while branch lengths are scaled according to the number of nucleotide substitutions per site. The tree is midpoint rooted for clarity only.

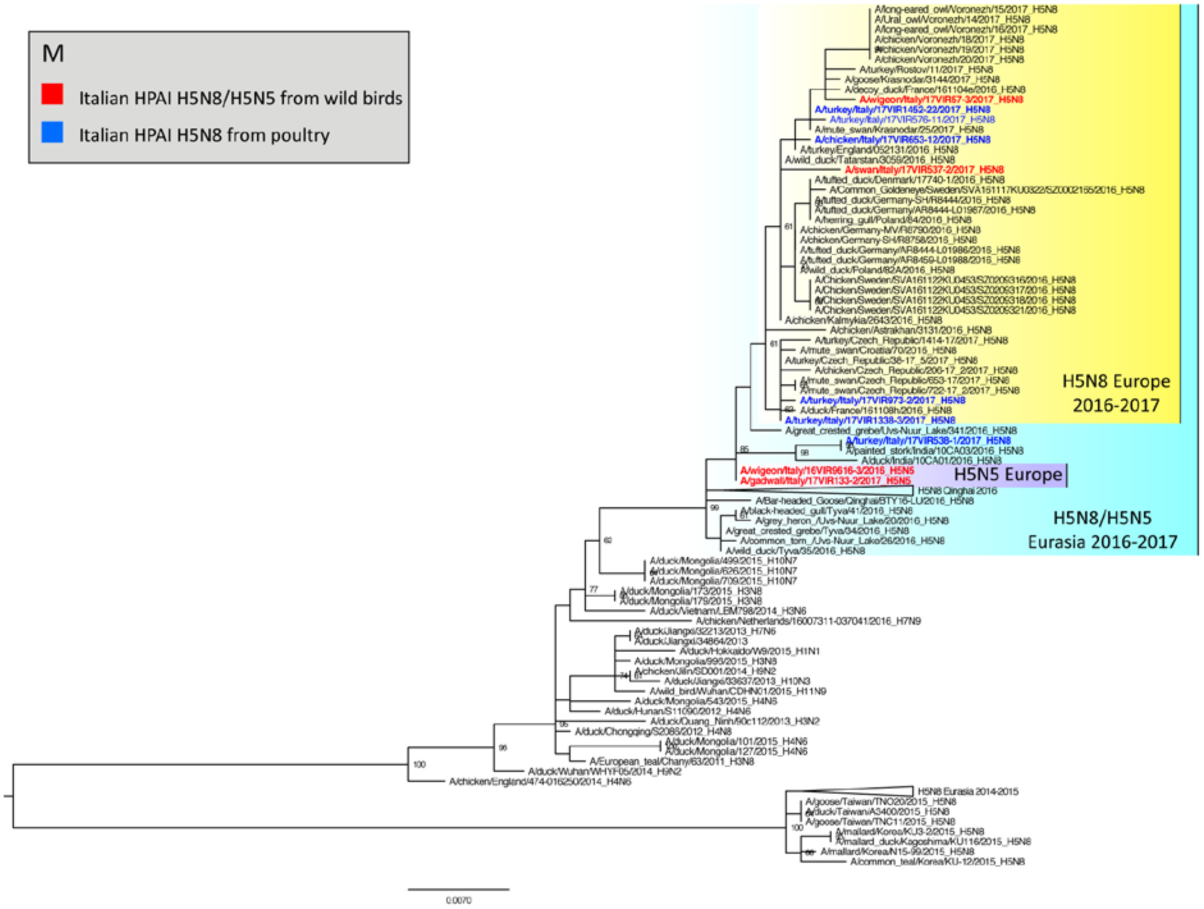

Technical Appendix Figure 7. Maximum Likelihood phylogenetic tree of the M gene. HPAI H5N8/H5N5 viruses were collected in Italy from wild (red) and domestic (blue) birds. Bootstrap supports higher than 60% are indicated next to the nodes, while branch lengths are scaled according to the number of nucleotide substitutions per site. The tree is midpoint rooted for clarity only.

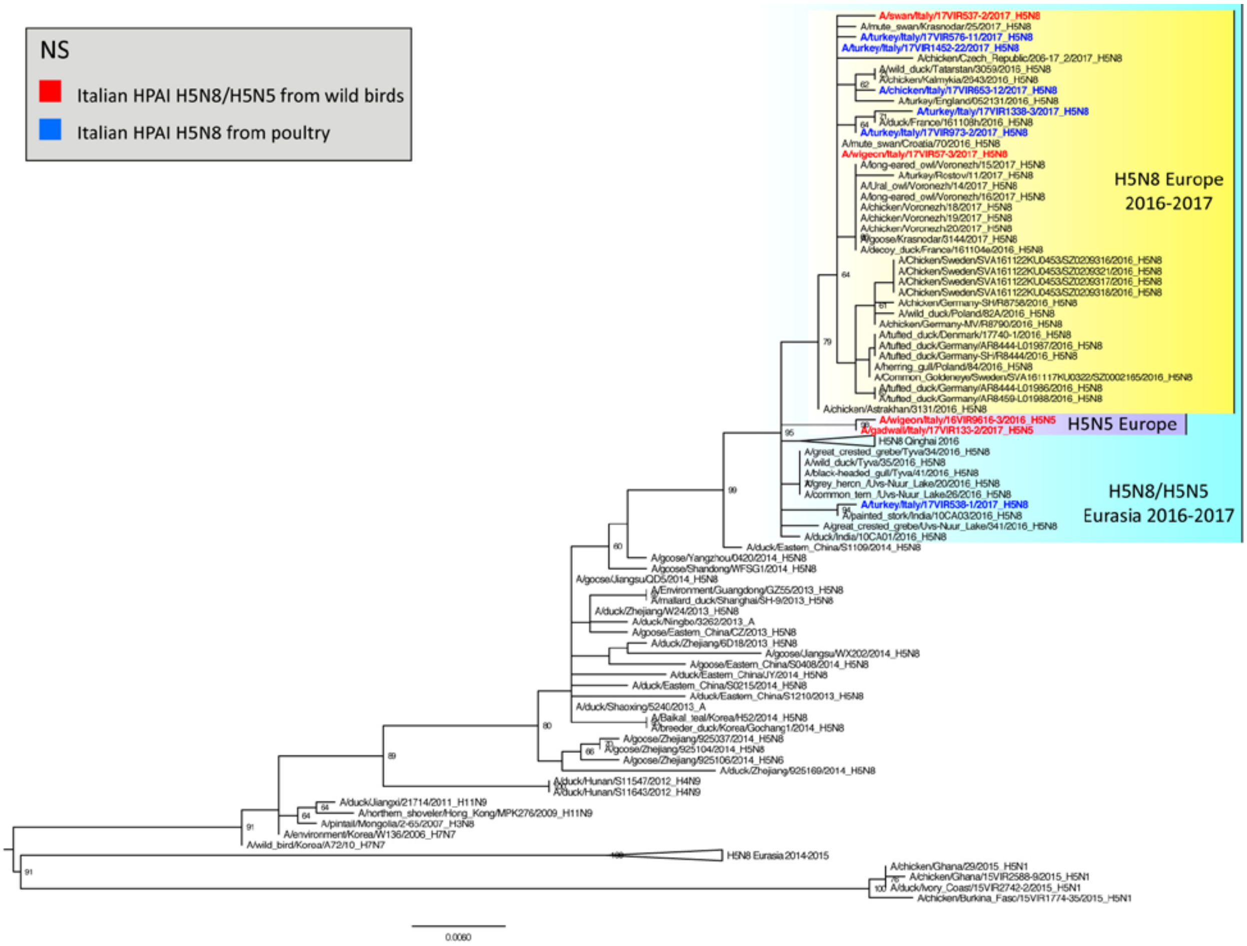

Technical Appendix Figure 8. Maximum Likelihood phylogenetic tree of the NS gene. HPAI H5N8/H5N5 viruses were collected in Italy from wild (red) and domestic (blue) birds. Bootstrap supports higher than 60% are indicated next to the nodes, while branch lengths are scaled according to the number of nucleotide substitutions per site. The tree is midpoint rooted for clarity only.

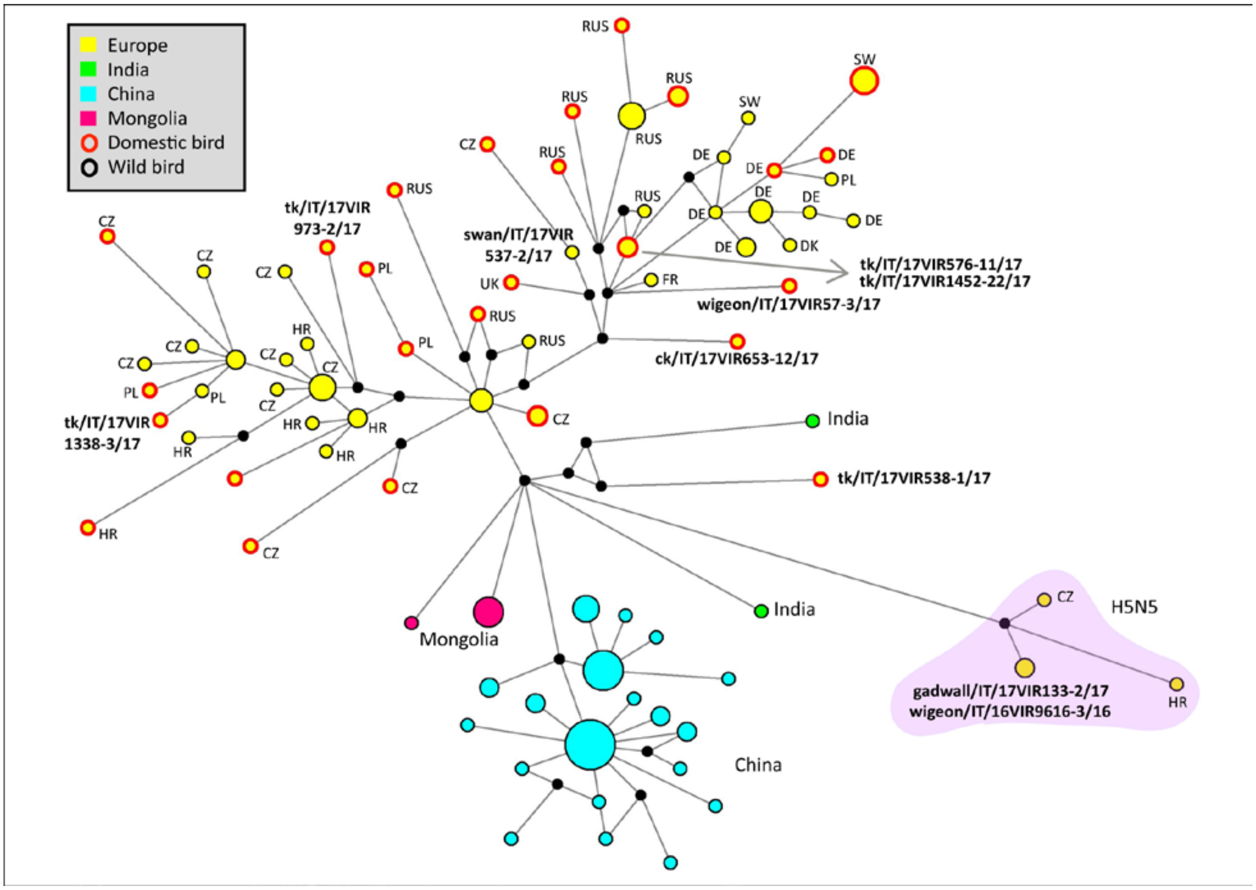

Technical Appendix Figure 9. Median-joining phylogenetic network of the HA gene sequences of the 2016 HPAI H5N8/H5N5 viruses from Eurasia. Each unique sequence genotype is represented by a circle sized relatively to its frequency in the dataset. Branches represent the shortest trees and are proportional to the number of nucleotide mutations that separate each node. Median vectors are indicated as black circles. The pink shading shows the H5N5 viruses.
